# Engineering *Clostridium* Strain to Accept Unmethylated DNA

**DOI:** 10.1371/journal.pone.0009038

**Published:** 2010-02-09

**Authors:** Hongjun Dong, Yanping Zhang, Zongjie Dai, Yin Li

**Affiliations:** 1 Institute of Microbiology, Chinese Academy of Sciences, Beijing, China; 2 Graduate School of the Chinese Academy of Sciences, Beijing, China; 3 Department of Biochemistry and Molecular Biology, University of Science and Technology of China, Hefei, China; The University of Manchester, United Kingdom

## Abstract

It is difficult to genetically manipulate the medically and biotechnologically important genus *Clostridium* due to the existence of the restriction and modification (RM) systems. We identified and engineered the RM system of a model clostridial species, *C. acetobutylicum*, with the aim to allow the host to accept the unmethylated DNA efficiently. A gene CAC1502 putatively encoding the type II restriction endonuclease Cac824I was identified from the genome of *C. acetobutylicum* DSM1731, and disrupted using the ClosTron system based on group II intron insertion. The resulting strain SMB009 lost the type II restriction endonuclease activity, and can be transformed with unmethylated DNA as efficiently as with methylated DNA. The strategy reported here makes it easy to genetically modify the clostridial species using unmethylated DNA, which will help to advance the understanding of the clostridial physiology from the molecular level.

## Introduction

Restriction and modification (RM) systems are widespread in bacteria and archea. Analyses of the available genome sequences of bacteria suggest that the bacterial RM systems are quite complex [1]. The occurrence of RM systems in prokaryotes has been explained as an ‘immune system’ to attack invading DNA that has not been properly modified (*e.g.* methylation) [2]. Most RM systems comprise a DNA methyltransferase (MTase) and a restriction endonuclease (REase). The MTase enables recognition of ‘self’ DNA by methylation of specific nucleotides within particular DNA sequences whereas the REase enzymatically cleaves the ‘foreign’ unmodified DNA [3]. Four groups of RM systems (types I, II, III, and IV) have been identified and described based on enzyme composition, cofactor requirements, and mode of action [4]. The RM system is a genetic barrier for gene transfer among bacteria or phage invasion [5]–[7]. Meanwhile, it is also a barrier for genetic manipulation such as conjugation [8] and transformation [9], which hampers the understanding and exploitation of many important bacteria through genetic manipulation.

*Clostridium* is one of the largest bacterial genera, ranking the second in size next to *Streptomyces*, and classified as Gram-positive endospore-forming obligate anaerobes [10]–[12]. Many species of *Clostridium* can cause severe diseases, for example, *C. botulinum* produces the toxin botulinum [13], *C. septicum* causes spontaneous gas gangrene or atraumatic myonecrosis [14], and *C. difficile* causes diarrhea and colitis [15]. On the other hand, some species of *Clostridium* are also of biotechnological importance [16], for instance *C. acetobutylicum* and *C. beijerinckii* used for solvent (acetone and butanol) production [17], and *Clostridium bifermentans* capable of degrading 2, 4, 6-trinitrotoluene (TNT), a toxic nitroaromatic explosive that accumulates in the environment [18]. Genetic manipulation is essential to understand the *Clostridium* strains so that strategies for either controlling or utilizing them can be developed. To date, genetic tools for targeted inactivation of genes have been reported for *Clostridium* strains, most notably in *C. acetobutylicum*, *C. beijerinckii*, *C. perfringens*, and most recently, in *C. difficile* [19]. The RM system seems to be a major barrier blocking the efficient genetic manipulation of *Clostridium* strains, which can be seen from the difficulties in conducting conjugation and transformation in a number of different clostridial species [20]–[23].

The objective of this study was to improve the genetic accessibility of clostridial species through manipulation of its RM systems. To this end, *C. acetobutylicum* was selected as a model clostridial species. The genome sequence of *C. acetobutylicum* ATCC824 has been determined [24], and several genetic manipulation methods specific to *C. acetobutylicum* have been developed. This includes a replication vector for gene overexpression [22], a gene inactivation system based on homologous recombination using replicable or nonreplicable vectors [25]–[29], and most recently the group II intron based gene inactivation system [30], [31]. Besides, the antisense RNA technology has also been employed in *C. acetobutylicum* [32], [33]. However, all these genetic manipulations require methylation of the target vector in a strain bearing pAN1 [34] or pAN2 [30] containing a gene Ф*3tI* encoding the Ф3TI methyltransferase from the phage Ф3TI of *Bacillus subtilis* [35]. The methylation step is necessary for transforming foreign DNA into *C. acetobutylicum* because of the presence of a type II restriction endonuclease Cac824I [22], which could recognize the GCNGC site. Although no research on cloning the gene encoding Cac824I has been reported, the REBASE has predicted that CAC1502 is the putative encoding gene based on genome information [1]. Some reports have indicated that the mutants of *B. subtilis* [36], *Thermosynechococcus elongatus* [37], and *Borrelia burgdorferi* [38] deficient in restriction endonuclease activity exhibited improved transformation efficiency. A genetically modified strain of *C. acetobutylicum* ATCC824 with disrupted *cac824I* has been reported [29]. However, the effect of *cac824I* disruption on clostridial RM activity and the consequent effect on the efficiency of genetic manipulation have not been reported. REBASE predicted that all sequenced 54 *Clostridium* strains belonging to 24 species have genes encoding restriction endonucleases [1]. Here we report that a CAC1502 disrupted mutant of *C. acetobutylicum* can happily accept unmethylated DNA. This might provide a general strategy to improve the genetic accessibility for clostridial species.

## Results

### Construction of a CAC1502 Disrupted Mutant *C. acetobutylicum* SMB009

**Figure 1:**
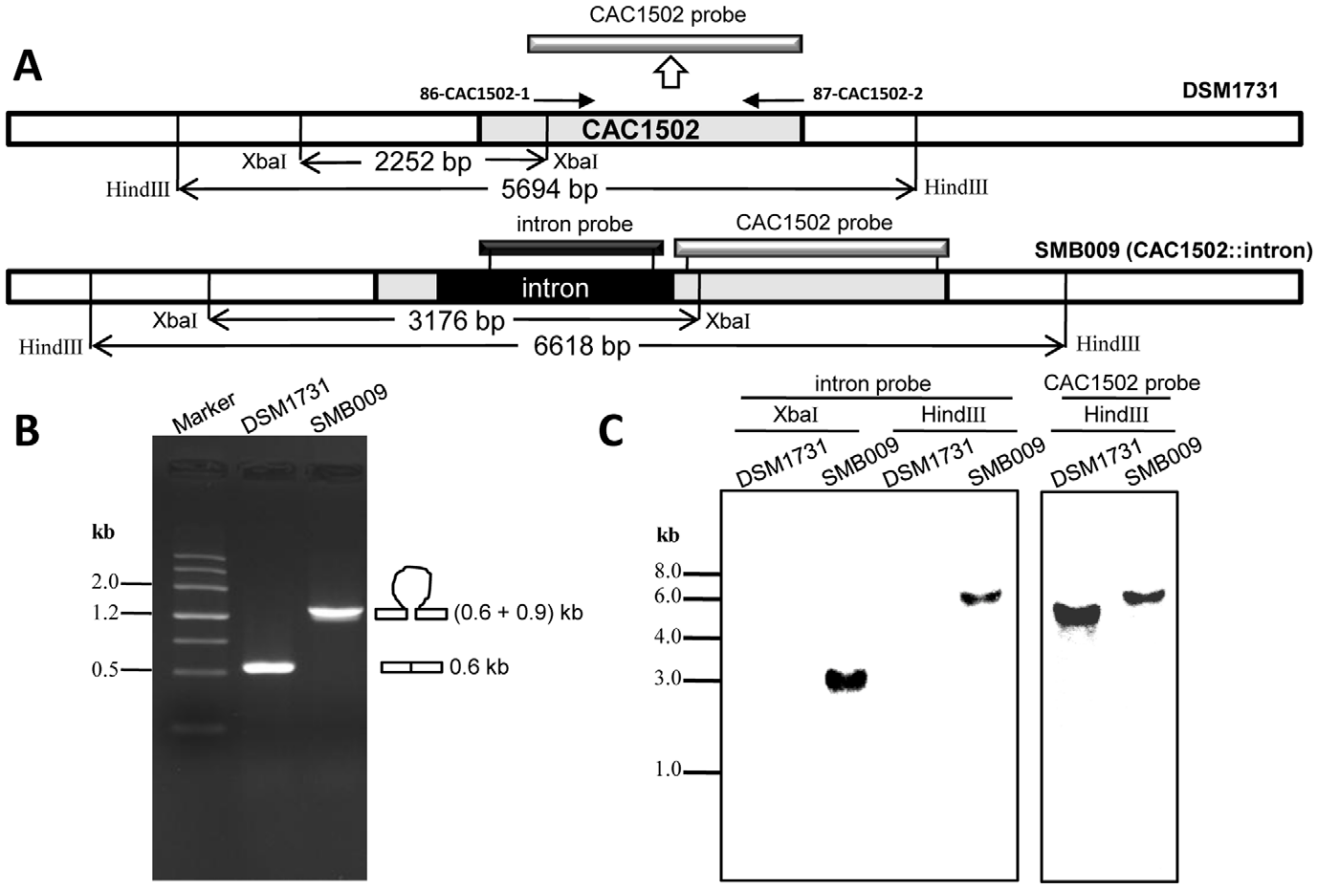
Construction of CAC1502 disrupted mutant *C. acetobutylicum* SMB009. **A.** Schematic show of the position of CAC1502 and the expected disrupted CAC1502 in the chromosome. 86-CAC1502-1 and 87-CAC1502-2 are the primers for generation of CAC1502 probe. **B.** Identification of the mutant SMB009 by PCR using primers 86-CAC1502-1 and 87-CAC1502-2 flanking the insertion site. **C.** Southern blot confirmation of intron insertion into CAC1502 using the intron probe and the CAC1502 specific probe.

To construct a CAC1502 disrupted mutant, a group II intron based system (ClosTron) [19] was used. Considering the antibiotic marker introduced by the ClosTron vector pMTL007 (GenBank: EF525477), we modified the vector to remove the RAM (Retrotransposition-Activated selectable Marker) [40] in order to construct a marker-free mutant. A potential intron insertion site 71/72a was predicted using the algorithm developed by Perutka [39]. Subsequently, a CAC1502 target vector pMTL007-CAC1502-71a was constructed based on pMTL007. The *lacI*-*traJ*-*oriT*-*fac* promoter fragment (2874 bp) was replaced by a thiolase promoter (141 bp) to remove the two MluI sites located at 9604 and 11809 in pMTL007. The resulted vector, designated as pMTL008, were digested by MluI and self-ligated to generate a vector pMTL009 without RAM. This vector was transformed into *C. acetobutylicum* DSM1731, and the CAC1502 disrupted mutant was screened according the reported procedure [30]. Among 900 colonies screened via PCR using the primers of 14-007-R1 and 87-CAC1502-2, one positive colony was obtained and designated as SMB009. The genomic structure of the mutant strain SMB009 was confirmed by Southern blot hybridization (Figure 1). The insertion of the intron into the target site was also confirmed by DNA sequencing (data not shown).

### No Activity of Cac824I Was Detected in Mutant SMB009

**Figure 2:**
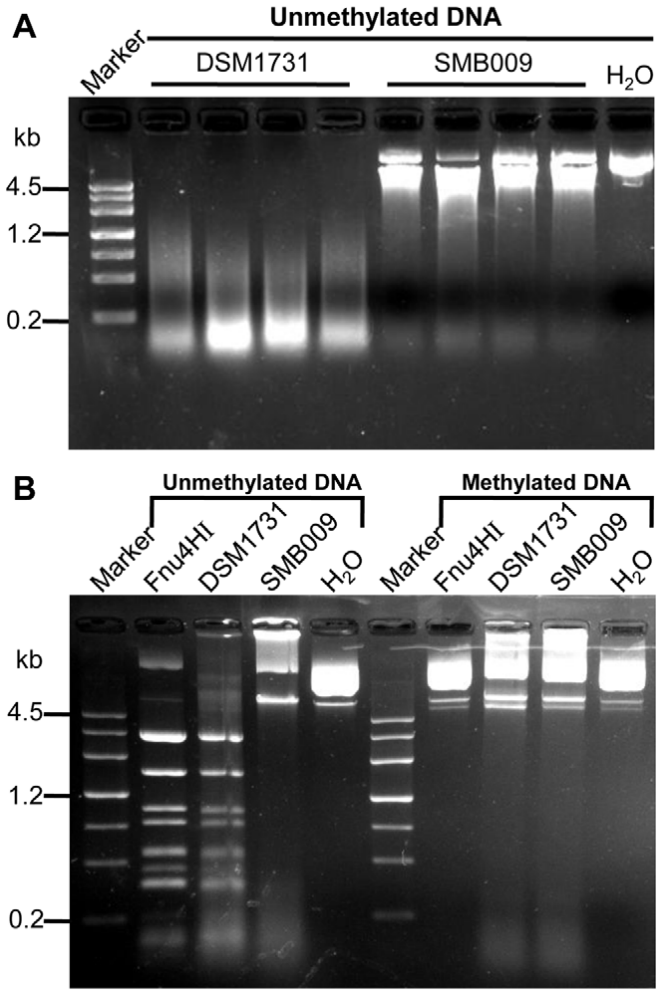
Detection of Cac824I activity in *C. acetobutylicum* mutant SMB009 using methylated or unmethylated pMTL007 as DNA substrate. **A.** Digestion of pMTL007 using the whole cell extracts of the wild type strain DSM1731 and the mutant SMB009 respectively. **B.** Digestion of pMTL007 using the protoplast extracts of the wild type strain DSM1731 and the mutant SMB009.

Although the gene CAC1502 in the genome of *C. acetobutylicum* has been predicted as the gene encoding Cac824I [22] by the REBASE [1], no experimental evidence has been reported to confirm the prediction. The activities of the type II restriction nuclease of the wild type strain DSM1731 and mutant SMB009 were investigated. The whole cell extract was used as the crude enzyme of Cac824I to analyze the nuclease activity using methylated or unmethylated pMTL007 as DNA substrate. The results showed that unmethylated pMTL007 was completely digested by the whole cell extract of the wild type strain DSM1731. In contrast, no significant digestion was observed when using the whole cell extract of the mutant SMB009 (Figure 2A), indicating difference in nuclease activities between the wild type and the mutant strains. Since it has been reported that there are non-specific nucleases located on the cell wall of *C. acetobutylicum* [22], we isolated the protoplast fractions of the wild type and the mutant strains, and compared the nuclease activities of the protoplast extracts of both strains. Figure 2B shows that the protoplast extract of the wild type strain DSM1731 exhibits the same digestion pattern with the control Fnu4HI, a restriction enzyme that recognizes the GCNGC site as Cac824I does. On the contrary, the protoplast extract of the mutant SMB009 cannot digest the tested DNA, indicating the absence of Cac824I activity.

### The Mutant SMB009 Could Efficiently Accept Unmethylated DNA

**Figure 3:**
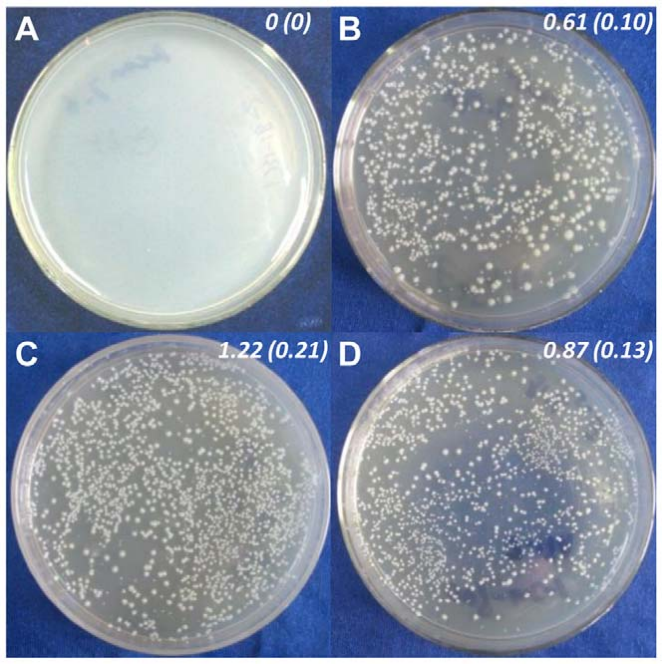
Electrotransformation performance of *C. acetobuytlicum* DSM1731 and SMB009 with methylated pIMP1 (pIMP1(m)) or unmethylated pIMP1. **A.** Strain DSM1731 transformed with pIMP1. **B.** Strain DSM1731 transformed with pIMP1(m). **C.** Strain SMB009 transformed with pIMP1. **D.** Strain SMB009 transformed with pIMP1(m). The italic numbers indicate corresponding transformation efficiency (10^5^ transformants/µg DNA) with standard deviations (n = 3) shown in parentheses. pIMP(m), methylated pIMP1 which was a mixture with pAN1.

The methylated and unmethylated shuttle vector pIMP1 [22] were transformed into the wild type strain DSM1731 and the mutant SMB009 to evaluate the effect of disrupting CAC1502 on the transformation performance of the host. The results showed that both *C. acetobutylicum* DSM1731 and SMB009 presented a similar efficiency to accept the methylated pIMP1, but only SMB009 could accept unmethylated pIMP1 as efficiently as accept the methylated DNA (Figure 3). For the mutant SMB009, the transformation efficiency of unmethylated pIMP1 is slightly higher than that of the methylated DNA (Figure 3). This might be due to that the methylated pIMP1 is a mixture of pIMP1 and pAN1, making it difficult to quantify the absolute amount of methylated pIMP1 in that mixture. Because the total DNA amount is considered as the pIMP1 amount in this work, the actual amount of methylated pIMP1 is less than the unmethylated DNA from *E. coli* ER2275.

### Genetic Manipulation of *C. acetobutylicum* SMB009 Using Unmethylated DNA

**Figure 4:**
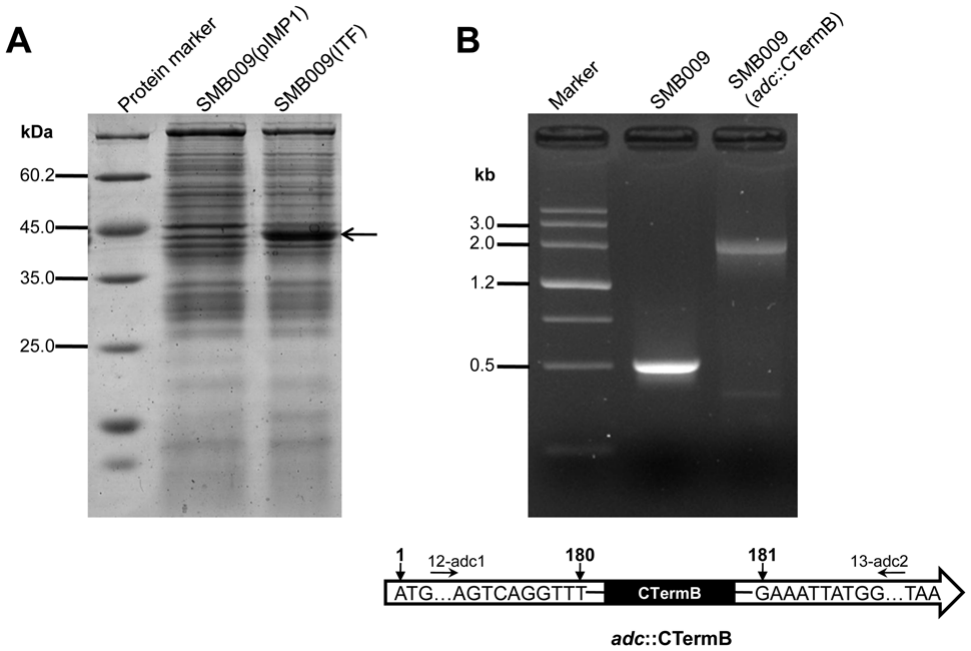
Genetic manipulation of *C. acetobutylicum* SMB009 using unmethylated DNA. **A.** Overexpression of *fdh* gene in *C. acetobutylicum* SMB009. The cells were grown to OD600 = 2∼3 in mRCM broth containing 50 µg/ml of erythromycin at 37°C. The boiled cell lysates were analyzed by SDS-PAGE (12% polyacrylamide gel). The overexpressed protein FDH (theoretical molecular weight 40 kDa predicted by DNAMAN Version 5) is indicated by the arrow on the right. **B.** Disruption of *adc* gene in *C. acetobutylicum* SMB009. Insertion of CTermB fragment into *adc* ORF was confirmed by PCR using the primers of 12-adc1 and 13-adc2 (corresponding to *adc* ORF positions 41-58 and 523-540 respectively). The insertion site of CTermB in *adc* ORF was validated by sequencing the PCR product of strain SMB009(*adc*::CTermB) amplified using the primers of 12-adc1 and 13-adc2.

To prove that *C. acetobutylicum* SMB009 can be genetically manipulated with unmethylated DNA, a recombinant plasmid pITF containing *fdh* gene from *Candida boidinii* (encoding formate dehydrogenase, GenBank: DQ458777) was isolated from *E. coli* JM109 without methylation, and transformed into *C. acetobutylicum* SMB009, resulting in SMB009(pITF). The expression of *fdh* gene in strain SMB009(pITF) was confirmed by SDS-PAGE (Figure 4A), suggesting a functional gene located on a unmethylated vector can be introduced into strain SMB009. Moreover, a ClosTron-based pMTL007-adc containing the modified intron targeting the *adc* gene (encoding acetoacetate decarboxylase) was constructed and isolated from *E. coli* JM109 without methylation. pMTL007-adc was electrotransformed into strain SMB009. The *adc* disrupted mutant SMB009(*adc*::CTermB) was identified by PCR with the primers of 12-adc1 and 13-adc2 flanking the insertion site (Figure 4B) and the insertion site was confirmed by sequencing the PCR product generated with primers of 12-adc1 and 13-adc2, and genomic DNA of SMB009(*adc*::CTermB) as template (Figure 4B). This suggests that genes in strain SMB009 can be inactivated using unmethylated vectors.

## Discussion

The very low efficiency of genetically modifying *Clostridium* strains has greatly hampered the progress for studying the physiology of this medically and biotechnologically important genus [19]. In this study, a mutant of *C. acetobutylicum* that can efficiently take up unmethylated DNA was obtained by only knocking out one gene CAC1502. Disruption of CAC1502 leads to the loss of the activity of a type II restriction nuclease, providing experimental evidence that CAC1502 encodes Cac824I as predicted by REBASE [1]. The feasibility of genetic manipulation in *C. acetobutylicum* SMB009 using unmethylated DNA was proved by overexpression of *fdh* gene from *C. boidinii*, and disruption of the native *adc* gene in SMB009 using unmethylated vectors. Strain SMB009 now has a foreign intron fragment (0.9 kb) on the chromosome. This does not affect making further mutants, as we have successfully inactivated the *adc* gene in strain SMB009 using pMTL007. However, the introduction of multiple ClosTron might result in repeated sequences in the genome, which might trigger homologous recombination event, although in a very low probability.

Cac824I is the third restriction nuclease found in clostridial species apart from CacI [41] and CacII [22]. REBASE [1] also predicts two other sets of RM systems in *C. acetobutylicum* ATCC824, one is CAC824ORF3534P RM system (Type II), and the other one is Cac824MrrP RM system (Type IV). In this work, no significant difference on the efficiency of transforming the methylated or unmethylated DNA into mutant SMB009 was observed, suggesting the Type II and Type IV RM systems might not be functional in *C. acetobutylicum*. In addition, no obvious degradation of DNA was observed when the DNA extracted from *E. coli* JM109 was digested by the whole cell extract of SMB009 (Figure 2), indicating that other predicted putative RM systems (except for Cac824I) did not show activities under experimental conditions.

To overcome the barrier effects of the RM systems on gene transfer, several strategies have been reported in the literatures. This includes:

1. Pre-modification of the vector before gene transfer [34], [42], [43]. The key to the success of this strategy is to select a proper methyltransferase, which is usually difficult due to the diversity and complexity of the RM systems in different hosts;
2. Using plasmids containing no or less restriction sites recognized by the RM systems of the host [8], [44]. The problem for this strategy is that reducing the restriction sites cannot be universally applied to different hosts;
3. Temporary inactivation of the RM systems under some abnormal conditions [45], [46]. Although sometimes effective, the loss of restriction function is always associated with loss of modification function, which means the phenotype will be reversed when the cells recovered from abnormal conditions;
4. Inactivation of the RM system [38], which is a clean and preferable method, but often limited by the availability of the genetic tools.

Thanks to the effective gene inactivation system ClosTron recently developed for *Clostridium* strains [19], we were able to disrupt the CAC1502 efficiently. This makes it easy to genetically modify the clostridial species using unmethylated DNA. REBASE has predicted 118 restriction enzymes encoding RM systems (including putative and function confirmed genes) in 54 *Clostridium* strains belonging to 24 clostridial species. The encoding genes for these restriction enzymes can be used as engineering targets with the aim to make *Clostridium* strains no longer to be a “genetically-tough” species to work with. This will help to advance the understanding of the clostridial physiology from the molecular level, and to expand the application of *Clostridium* strains for medical and biotechnological purposes.

## Materials and Methods

### Bacterial Strains, Plasmids, and Primers

Bacterial strains and plasmids used in this work are listed in Table 1, and the primers are listed in Table 2. *E. coli* JM109 was routinely used for vectors construction, *E. coli* TOP10 bearing pAN2 for methylation of pMTL007 deprived plasmids, and *E. coli* ER2275 bearing pAN1 for methylation of pIMP1. All primers were synthesized by Invitrogen (Beijing, China) followed by polyacrylamide gel electrophoresis purification.

**Table 1:** Bacterial strains and plasmids.

Strains or plasmids	Relevant characteristics	Reference or source
Strains		
*E. coli* TOP10	*mcrA Δ(mrr-hsdRMS-mcrBC) recA1*	Invitrogen
*E. coli* JM109	*recA1 mcrB^+^ hsdR17*	Lab storage
*E. coli* ER2275	*recA mcrBC hsdR*	NEB
*C. acetobutylicum* DSM1731	Contains CAC1502, wild type	DSMZ
*C. acetobutylicum* SMB009	CAC1502::intron	This work
Plasmids		
pMTL007	Cm^R^, ClosTron	[30]
pAN2	Ф*3tI*, *p15a ori*, Tet^R^	[30]
pMTL007-CAC1502-71a	Derived from pMTL007, targeting the CAC1502 in *C. acetobutylicum*	This work
pMTL008	Deprived from pMTL007-CAC1502-71a, replacement of the *lacI-traJ-oriT-fac* promoter fragment by Pthl	This work
pMTL009	Deprived from pMTL008, loss of ErmBtdRAM1	This work
pIMP1	MLS^R^ Ap^R^ shuttle vector of *E. coli*-*C. acetobutylicum*	[22]
pAN1	Ф*3tI*, *p15a ori*, Cm^R^	[22]
pITF	*fdh* expression vector	This work
pMTL007-adc	*adc* disrutption vector	This work

**Abbreviations:** Ap^R^, ampicillin resistance; Cm^R^, chloramphenicol resistance; MLS^R^, macrolide, lincosamide, and streptogramin B resistance; Tet^R^, tetracycline resistance; Ф*3tI*, Ф3TI methyltransferase gene of *Bacillus subtilis* phage Ф3TI. Pthl, thiolase gene promoter (Pthl) from *C. acetobutylicum;* DSMZ, German Collection of Microorganisms and Cell Cultures, Braunschweig, Germany; NEB, New England Biolabs Beverly, MA.

**Table 2:** Primers used in this study.

Primers	Sequence (5′—3′)	Source
10-EBS Universal	CGAAATTAGAAACTTGCGTTCAGTAAAC	[30]
11-5402F-F1	TTAAGGAGGTGTATTTCATATGACCATGATTACG	[30]
14-007-R1	AGGGTATCCCCAGTTAGTGTTAAGTCTTGG	[30]
86-CAC1502-1	AATGCAGTATCAGTACCAATTATAAG	This work
87-CAC1502-2	AACTTATTAAATTCAGCCACCTTTTG	This work
92-CAC150271/72a-IBS	AAAAAAGCTTATAATTATCCTTACTTAATGATCCAGTGCGCCCAGATAGGGTG	This work
93-CAC1502-71/72a-EBS1d	CAGATTGTACAAATGTGGTGATAACAGATAAGTCGATCCAAATAACTTACCTTTCTTTGT	This work
94-CAC1502-71/72a-EBS2	TGAACGCAAGTTTCTAATTTCGGTTTTAAGTCGATAGAGGAAAGTGTCT	This work
95-IBS(CAC1502)	TACTTAATGATCCAG	This work
151-Pthl-1(007)	AGTCGGCGCCTATATTGATAAAAATAATAATAGTGGG	This work
152-Pthl-2(007)	ATGCAAGCTTTCTAACTAACCTCCTAAATTTTG	This work
153-Intron-Probe1	GTTATCACCACATTTGTACAA	This work
154-Intron-Probe2	ACGCGTCGCCACGTAATAAATATC	This work
163-CAC1502-probe1	TGGATCATTAAGCGGTCTATTTAAAG	This work
164-CAC1502-probe1	CTAAAGTTGTTCCGGAATCTTAATC	This work
Pthl1	AGTGTCGACTATATTGATAAAAATAATAATAGTGGG	This work
Pthl2	CGTGGATCCTTCTTTCATTCTAACTAACCTCC	This work
fdh1	CGTGGATCCATGAAGATCGTTTTAGTC	This work
fdh2	GCGGAATTCTTATTTCTTATCGTGTTTAC	This work
1-adc180/181s-IBS	AAAAAAGCTTATAATTATCCTTATTAGTCAGGTTTGTGCGCCCAGATAGGGTG	This work
2-adc180/181s-EBS1d	CAGATTGTACAAATGTGGTGATAACAGATAAGTCAGGTTTGATAACTTACCTTTCTTTGT	This work
3-adc180/181s-EBS2	TGAACGCAAGTTTCTAATTTCGGTTACTAATCGATAGAGGAAAGTGTCT	This work
12-adc1	TAACTTCGCCTGCATTTC	This work
13-adc2	TATTCTAGGGCTTCCATC	This work

### Growth and Maintenance Conditions

*Escherichia coli* strains were grown aerobically at 37°C in Luria-Bertani medium supplemented, when necessary, with ampicillin (100 µg/ml), chloramphenicol (30 µg/ml), or tetracycline (40 µg/ml). All *C. acetobutylicum* strains were grown anaerobically at 37°C in different media supplemented, when necessary, with chloramphenicol (30 µg/ml) or erythromycin (25 µg/ml). *C. acetobutylicum* strains were grown in RCM medium [47] for routine growth, and mRCM medium (RCM containing 20 g/L glucose as the sole carbohydrate) for making competent cells. All *C. acetobutylicum* and *E. coli* strains were maintained frozen in 15% glycerol at −80°C.

### DNA Isolation and Manipulation

Total genomic DNA of *C. acetobutylicum* and the plasmid DNA of *E. coli* were prepared using an E.Z.N.A Bacterial DNA Isolation Kit and E.Z.N.A Plasmid Extraction Kit (Omega Biotek Inc., Guangzhou, China), respectively. DNA restriction and cloning were performed according to standard procedures [48]. Restriction enzymes and T4 DNA ligase were obtained from New England BioLabs (Beijing, China), and used according to the manufacturer's instructions.

### Construction of pMTL009

**Figure 5:**
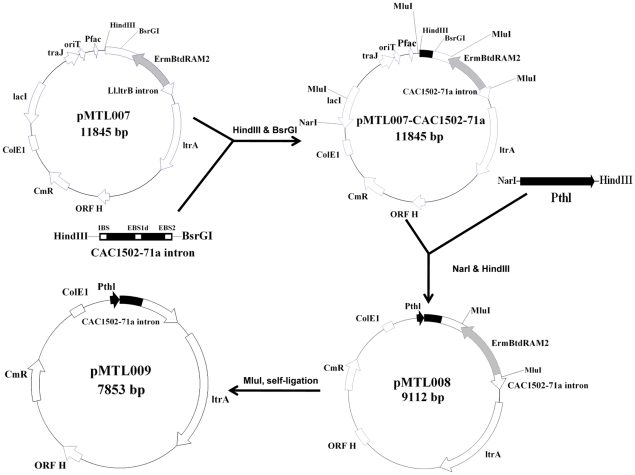
Schematic representation of the construction of the pMTL009 for CAC1502 disruption.

*E. coli* JM109 was used for construction of pMTL009. Firstly, target site for disruption of CAC1502 was selected and the intron re-targeting PCR primers (*i.e.*, 92-CAC1502-71/72a-IBS, 93-CAC1502-71/72a-EBS1d, and 94-CAC1502-71/72a-EBS2) were designed according to a computer algorithm [39]. One-tube SOEing PCRs were used to amplify and assemble the 353 bp PCR product which contains the modified IBS, EBS1d and EBS2 sequences that are responsible for intron targeting. PCR was performed according to the Targetron Gene Knockout System kit Protocol (http://www.sigmaaldrich.com). The targeting region was cloned into pMTL007 [30] to replace the original intron fragment for generation of pMTL007-CAC1502-71a, using 95-IBS (CAC1502) and 14-007-R1 to screen for positive clones containing pMTL007-CAC1502-71a. The structure of the plasmid was confirmed by DNA sequencing using primer 11-5402F-F1. Secondly, the CAC1502 target plasmid pMTL007-CAC1502-71a was digested by HindIII and NarI, to remove the 2507 bp fragment containing the *fac* promoter [30] and the two MluI sites. The thiolase gene promoter (141 bp, Pthl) was amplified from the genomic DNA of *C. acetobutylicum* using primers 151-Pthl-1(007) and 152-Pthl-2(007). The resulted PCR product containing the HindIII and NarI sites was digested and ligated to the HindIII and NarI digested pMTL007-CAC1502-71a to generate pMTL008. Finally, pMTL008 was digested by MluI to remove the ErmBtdRAM1 [30] fragment, and self-ligated to generate pMTL009 (Figure 5).

### Electrotransformation and Screening for Mutants

Prior to transformation into *C. acetobutylicum*, plasmids were methylated in the corresponding *E. coli* host (see Table 1 and Table 2) when necessary. Electrotransformation of *C. acetobutylicum* was carried out according to the protocol developed by Mermelstein [22]. *C. acetobutylicum* SMB009 was screened according to the protocol reported by Heap [30]. Because SMB009 has no antibiotic marker, the DSM1731 bearing the ClosTron vector pSMB009 was firstly plated on the RCM plate without antibiotic to form colonies. The colonies were then streaked on new RCM plates without antibiotics (30 colonies/plate). The clones grown on one plate were pooled into one 1.5 ml EP tube, and the genomic DNA was isolated and used as template for PCR using primers 10-EBS Universal (located on intron) and 87-Cac824I-2 (located on chromosome). If the PCR result is positive, each clone on that plate will be tested by PCR until the positive clone was obtained.

### Construction of *fdh* Overexpression Strain SMB009(pITF) and *adc*-Disrupted Strain SMB009(*adc*::CTermB)

To construct the *fdh* gene (encoding formate dehydrogenase, GenBank: DQ458777) expression vector pITF, pIMP1 was used as parent vector. *fdh* was amplified from the genomic DNA of *Candida boidinii* using the *fdh1* primer and *fdh2* primer containing BamHI and EcoRI restriction sites respectively. The promoter region of thiolase gene (Pthl) was amplified from the genomic DNA of *C. acetobutylicum* DSM1731 using Pthl1 primer and Pthl2 primer containing SalI and BamHI restriction sites respectively. The mixture of pIMP1, PCR products of *fdh* and Pthl were digested by EcoRI, BamHI and SalI in NEB Buffer 3. The digested products were purified by an E.Z.N.A Cycle-Pure Kit (Omega Biotek Inc., Guangzhou, China), and then ligated together by T4 DNA ligase to generated pITF. The pITF construct was confirmed by sequencing. For disruption of *adc* gene (encoding acetoacetate decarboxylase, NCBI-GeneID: 1116170) in SMB009, the vector pMTL007-adc was constructed. In the ORF of *adc* gene, the site of 180/181 (A from the initial code ATG was marked as 1) was selected as the insertion site of intron (*i.e.* CTermB from pMTL007) according to a computer algorithm [39]. The corresponding primers, *i.e.* 1-adc180/181s-IBS, 2-adc180/181s-EBS1d, 3-adc180/181s-EBS2 were designed to construct pMTL007-adc as construction of pMTL007-CAC1502-71a. After electrotransformation of pMTL007-adc into strain SMB009, the transformants (chloramphenicol resistant colonies) were restreaked on the RCM plate containing 50 µg/ml of erythromycin to screen for integrants. The genomic DNA of erythromycin resistant colonies was extracted for PCR confirmation, using the primers of 12-adc1 and 13-adc2 (corresponding to *adc* ORF positions 41-58 and 523-540 respectively), to screening for *adc*-disrupted mutants (designated as SMB009(*adc*::CTermB)). The vector pMTL007-adc in SMB009(*adc*::CTermB) was cured by subculturing in RCM without antibiotics.

### Southern Blot Analysis

The genomic DNA of the wild type strain *C. acetobutylicum* DSM1731 and CAC1502 disrupted mutant SMB009 were prepared using the E.Z.N.A Bacterial DNA Isolation Kit (Omega Biotek Inc., Guangzhou, China). For Southern hybridization, DNAs were digested with restriction enzymes, run in 1.0% agarose gel, blotted to a positively charged nylon transfer membrane (Amersham HybondTM-N+, GE Healthcare, Little Chalfont, Buckinghamshire, UK), and hybridized with a DIG-labeled probe corresponding to CTermB positions 294-696 [49]. The intron specific probe for DIG-DNA labeling was generated by PCR using pMTL007 as a template and primers 153-Intron-Probe1 and 154-Intron-Probe2. The CAC1502 specific probe for DIG-DNA labeling was obtained by PCR using the genomic DNA of the wild type strain DSM1731 as a template and primers 163-CAC1502-probe1 &164-CAC1502-probe1. The process of Southern analysis was carried out using the DIG High Prime DNA Labeling and Detection Starter Kit I (Roche Diagnosis GmbH, Roche Applied Science, 68298 Mannhein Germany) according to the instruction manual.

### Preparation of Crude Proteins for the Detection of Cac824I

The proteins of whole cell were extracted from the cells (OD600 = 0.6, cultured in 100 ml mRCM medium) lysed by sonication for 30 min at a power level of 200 W, in 15 ml distilled water. The protoplast extract (15 ml) were extracted from the cells (OD600 = 0.6, cultured in 100 mRCM containing 0.4% glycine), following a protocol developed by Mermelstein [22]. The crude proteins were used to detect the activity of Cac824I employing pMTL007 [30] (methylated or unmethylated) as DNA substrate. The general reaction system contains 1 µg DNA, 5 µl crude proteins and 1×NEB Buffer 4 (NEB) in volume of 20 µl. The reaction conditions are 37°C for 8 hours.

### SDS-PAGE

Transformant of *C. acetobutylicum* SMB009(pITF) and plasmid control strain SMB009(pIMP1) were inoculated into 10 ml mRCM medium (see Growth and maintenance conditions) plus erythromycin (50 µg/ml) and grown to OD600 2–3 at 37°C. 1.5 ml of the culture was centrifuged to harvest the cells, which was then resuspended in 0.1 ml of SDS-PAGE loading buffer. After boiling for 5 min, samples (20 µl) were applied to a SDS-PAGE (sodium dodecyl sulfate– polyacrylamide gel, 12%) and visualized by Coomassie blue staining. The gel was scanned by ImageScanner 3 (GE Healthcare).
